# Predictive Model of Type 2 Diabetes Remission after Metabolic Surgery in Chinese Patients

**DOI:** 10.1155/2020/2965175

**Published:** 2020-10-31

**Authors:** Yufang Luo, Zi Guo, Honghui He, Youbo Yang, Shaoli Zhao, Zhaohui Mo

**Affiliations:** Department of Endocrinology, Third Xiangya Hospital of Central South University & Diabetic Foot Research Center of Central South University, Changsha, Hunan Province 410013, China

## Abstract

**Introduction:**

Metabolic surgery is an effective treatment for type 2 diabetes (T2D). At present, there is no authoritative standard for predicting postoperative T2D remission in clinical use. In general, East Asian patients with T2D have a lower body mass index and worse islet function than westerners. We aimed to look for clinical predictors of T2D remission after metabolic surgery in Chinese patients, which may provide insights for patient selection.

**Methods:**

Patients with T2D who underwent metabolic surgery at the Third Xiangya Hospital between October 2008 and March 2017 were enrolled. T2D remission was defined as an HbA1c level below 6.5% and an FPG concentration below 7.1 mmol/L for at least one year in the absence of antidiabetic medications.

**Results:**

(1) Independent predictors of short-term T2D remission (1-2 years) were age and C-peptide area under the curve (C-peptide AUC); independent predictors of long-term T2D remission (4–6 years) were C-peptide AUC and fasting plasma glucose (FPG). (2) The optimal cutoff value for C-peptide AUC in predicting T2D remission was 30.93 ng/ml, with a specificity of 67.3% and sensitivity of 75.8% in the short term and with a specificity of 61.9% and sensitivity of 81.5% in the long term, respectively. The areas under the ROC curves are 0.674 and 0.623 in the short term and long term, respectively. (3) We used three variables (age, C-peptide AUC, and FPG) to construct a remission prediction score (ACF), a multidimensional 9-point scale, along which greater scores indicate a better chance of T2D remission. We compared our scoring system with other reported models (ABCD, DiaRem, and IMS). The ACF scoring system had the best distribution of patients and prognostic significance according to the ROC curves.

**Conclusion:**

Presurgery age, C-peptide AUC, and FPG are independent predictors of T2D remission after metabolic surgery. Among these, C-peptide AUC plays a decisive role in both short- and long-term remission prediction, and the optimal cutoff value for C-peptide AUC in predicting T2D remission was 30.93 ng/ml, with moderate predictive values. The ACF score is a simple reliable system that can predict T2D remission among Chinese patients.

## 1. Introduction

The number of patients with type 2 diabetes (T2D) and obesity is increasing worldwide. According to the latest epidemiology reports, the prevalence of T2D in China has risen to 10.4% [1]. Furthermore, it is predicted that by 2025, more than 60% of patients with T2D worldwide will be in Asian countries [2]. Currently, usual therapy for most patients with T2D and obesity is lifestyle and pharmacological interventions. In recent decades, metabolic surgery (MS) has been widely accepted as a method of treatment for T2D. Several clinical randomized controlled trials indicated that, in comparison to conventional therapy, MS is not limited to improving obesity and T2D and effectively alleviates comorbidities such as hypertension and dyslipidemia, thus reducing cardiovascular-related mortality [3–9]. The mechanisms underlying MS for the treatment of T2D include food restriction, promotion of incretin secretion, changes in bile acid composition, and changes in intestinal microbiota [10–13]. The most common surgical procedures are gastrointestinal bypass (GB) and sleeve gastrectomy (SG) [14]. The development of perioperative care and laparoscopic techniques guarantees the safety of metabolic surgery, which has been adopted by T2D prevention guidelines in numerous countries.

As MS remains the most effective treatment for obesity and T2D, several studies have investigated the factors affecting T2D outcomes after surgery. Studies have demonstrated that race, age, duration of T2D, weight, body mass index (BMI), glycosylated hemoglobin (HbA1c) level, fasting plasma glucose (FPG) level, fasting C-peptide level (FCP) concentration, preoperative insulin use, and hypertension may affect the outcomes of MS [15–23]. Recently, scholars attempted to construct models [24, 25] and scoring systems to predict the resolution of T2D after bariatric surgery, such as the ABCD (age, BMI, C-peptide, and duration of T2D) [26], Integrated Medical Services (duration of T2D, preoperative number of diabetes medications, insulin use, and HbA1c) [27], DiaRem (age, HbA1c, antidiabetic drugs, and insulin treatment) [28], and Diabetes Risk Score (age, duration of T2D, BMI, microvascular and macrovascular complications, insulin use, and stimulated C-peptide level) [29], to predict the resolution of T2D after bariatric surgery. Variations in these reports may be due to differences in designs, populations, follow-up times, and operation procedures, and no authoritative standard for prediction is yet available for clinical use.

Previous reports have shown that East Asian patients with newly diagnosed T2D have lower BMIs and worse *β*-cell function than Western patients [30]. According to recently released guidelines, the eligibility for bariatric surgery in Asian patients with T2D, which is based on BMI, should be lowered to 27.5 kg/m^2^ [31, 32]. However, the islet function index has not been included in the selection of MS indications. Although numerous studies have emphasized the value of *β*-cell function in T2D remission prediction [22, 23, 26, 33], only a limited number of studies have focused on comprehensive indexes of islet function [34]. To providing a better understanding of *β*-cell function in the remission of Chinese patients with T2D, we performed a study applying various indicators that reflect islet function. Our study aimed to explore the predictive factors for T2D remission in the short term and long term after MS and to further establish a remission prediction score system based on mainland Chinese patients. A better understanding of the predictors for MS will help guide the choice of T2D therapy.

## 2. Materials and Methods

### 2.1. Patient Population

This prospective cohort study included 87 patients with T2D undergoing MS as inpatients in the Department of Endocrinology between October 2008 and March 2017. T2D was defined according to the 1999 World Health Organization (WHO) diagnostic criteria for T2D or the preoperative use of diabetic medication. The inclusion criteria were as follows: (1) age between 18 and 70 years; (2) before May 2011, BMI ≥24 kg/m^2^ (before the promulgation of the Chinese guidelines for T2D), and BMI ≥27.5 kg/m^2^ thereafter; and (3) clinical diagnosis of T2D for no more than 15 years. The exclusion criteria were as follows: (1) abuse of alcohol or drugs or uncontrollable mental illness; (2) severe organ dysfunction or other diseases related to surgery intolerance; (3) combined acute complications such as diabetic ketoacidosis and a hyperosmolar hyperglycemic state; and (4) patients with type 1 diabetes, gestational diabetes, or other types of diabetes. The study was approved by the Third Xiangya Hospital Ethics Committee.

### 2.2. Data Collection

Data were obtained from the outpatient and inpatient departments. Age, duration of T2D, blood pressure, glucose, C-peptide, insulin, HbA1c, urinary microalbumin (UmAlb), and lipid biomarkers were documented before surgery and at the follow-up visits. Loss to follow-up was defined by no current available contact information or no response to three-time inquiries by phone. The main reasons for failing to participate in the follow-ups included an incorrect phone number, unaffordable medical expenses, migration, and long distance.

### 2.3. Indexes of Islet Function

This study included multiple indexes of *β*-cell function [35], including fasting C-peptide (FCP), C-peptide area under the curve (C-peptide AUC), first- and second-phase insulin secretion indexes (ΔC30 and ΔC120), insulinogenic indexes (ΔC30/ΔG30 and ΔC120/ΔG120), homeostasis model assessment of *β*-cell function (HOMA-*β*), and homeostasis model assessment of insulin resistance (HOMA-IR). To eliminate the influence of exogenous insulin use, we used C-peptide as a substitute for insulin. The calculation of HOMA-*β* and HOMA-IR was proposed in a previous paper [36]. The formulas were as defined as follows:

(1) $$\begin{array}{c} \Delta C30=\text{C}-\text{peptide}\left(30\text{ min}\right)–\text{FCP,}\\ \Delta C120=\text{C}-\text{peptide}\left(120\text{ min}\right)–\text{FCP,}\\ \frac{\Delta C30}{\Delta G30}=\frac{\Delta C30}{\left[\text{glucose}\left(30\text{ min}\right)–\text{FPG}\right]},\\ \frac{\Delta C120}{\Delta G120}=\frac{\Delta C120}{\left[\text{glucose}\left(120\text{ min}\right)–\text{FPG}\right]},\\ \text{HOMA}-\beta \left(\text{CP},\text{DM}\right)=0.27\times \frac{\text{FCP}}{\left(\text{FPG}–3.5\right)}\text{\%,}\\ \text{HOMA}-\text{IR}=\frac{1.5+\text{FCP}\times \text{FPG}}{2800},\\ \text{C}-\text{peptide }AUC=0.25\times \text{FCP}+4\times \text{C}-\text{peptide}\left(30\text{ min}\right)+3\times \text{C}-\text{peptide}\left(120\text{ min}\right). \end{array}$$

### 2.4. Outcome Evaluation

T2D remission was defined as an HbA1c level below 6.5% and an FPG concentration below 7.1 mmol/L for at least one year in the absence of antidiabetic medications. Recurrence was defined as the return of FPG or HbA1c levels to the diabetic range (≥7.0 mmol/L and ≥6.5%, respectively) or the need for antidiabetic medication after initial remission. Short-term remission was defined as T2D remission in the first 1-2 years after surgery, and long-term remission was defined as T2D remission for 4–6 years after surgery.

### 2.5. Scoring System

#### 2.5.1. ABCD Score

Four parameters, including age, BMI, C-peptide level, and duration of T2D, were used for scoring (total points ranged from 0 to 10) [26]. One point was used for age. Each of the remaining three variables had 4 categories, and 0–3 points were assigned. Patients with higher ABCD scores were predicted to have a higher probability of T2D remission after surgery.

#### 2.5.2. DiaRem Score

Four parameters, including age, HbA1c, anti-T2D drugs, and insulin, were used for scoring (total points ranged from 0 to 22) [28]. The age score ranged from 0 to 3, and HbA1c ranged from 0 to 6 due to different categories. A 3-point score was used for using anti-T2D drug use, and a 10-point score was used for insulin use. Patients with lower DiaRem scores were predicted to have a higher probability of T2D remission after surgery.

#### 2.5.3. Individualized Metabolic Surgery (IMS) Score

Four parameters, including number of T2D medications, insulin use, T2D duration, and HbA1c, were used for scoring [27]. A constructed nomogram allowed for the classification of diabetes severity into mild, moderate, and severe groups. Patients with lower IMS scores were predicted to have a higher probability of T2D remission after surgery.

### 2.6. Statistical Analysis

All analyses were performed using IBM SPSS for Windows, version 23.0 (IBM Corp., Armonk, NY). Continuous variables were expressed as the means with SD or with median. Baseline comparisons were performed using chi-square tests and *t*-tests. Relationships between two continuous variables were tested by Pearson correlation analysis. Univariate regression analysis was used to screen for possible predictors, and binary regression analysis was used to identify the independent predictors of T2D remission. Receiver operating characteristic (ROC) curve analysis was performed to determine the diagnostic value, and the optimal cutoff was specified with Youden's J index (=sensitivity + specificity−1). All statistical tests were tested two sided, and *P* < 0.05 was considered statistically significant.

## 3. Results

### 3.1. Baseline Characteristics

This study included a total of 87 patients with T2D (60 males and 27 females). The average age of the patients was 44.2 ± 11.2 years, and the T2D duration was 6.4 ± 4.8 years. The average patients' BMI was 31.29 ± 6.51 kg/m^2^. There were 9 patients (10.3%) taking lifestyle interventions without medicine before surgery, 32 patients (36.8%) using oral antidiabetic drugs, 20 patients (23.0%) using insulin, and 26 patients (29.9%) using oral antidiabetic drugs combined with insulin, respectively. GB was performed in 62 patients, while the others received SG. The patients' baseline data are shown in Table 1. Patients who underwent SG were younger and heavier and had better residual *β*-cell function than the patients who underwent GB (*P* < 0.05).

Here, we analysed the relationship between the course of disease and *β*-cell function indexes. Duration of T2D was negatively correlated with FCP (*r* = −0.257, *P*=0.016), ΔC30/ΔG30 (*r* = −0.279, *P*=0.011), HOMA-IR (CP) (*r* = − 0.270, *P*=0.012), and C-peptide AUC (*r* = −0.257, *P*=0.020). No correlation was observed between duration of T2D and ΔC30 (*r* = − 0.149, *P*=0.181), ΔC120 (*r* = −0.138, *P*=0.202), ΔC120/ΔG120 (*r* = −0.067, *P*=0.535), and HOMA-*β* (CP, DM) (*r* = −0.071, *P*=0.515).

### 3.2. Predictors of Short-Term T2D Remission

A total of 53 of the 87 (60.92%) patients achieved T2D remission at 1-2 years of follow-up. Among them, there were 23 patients who underwent SG and 30 patients who underwent GB, respectively. Univariate regression showed that patients with a younger age, higher BMI, shorter duration of T2D, SG, no presurgical insulin use, higher levels of FCP, second-phase insulin secretion indexes (ΔC120), HOMA-*β* (CP, DM), HOMA-IR (CP), and C-peptide AUC values (the values of *β*-cell function indexes were grouped by median) had higher T2D remission rates than other patients 1-2 years after surgery (Table 2). There was no significant difference in remission rates among groups classified by sex, lipid profiles, umAlb, HbA1c, FPG, first-phase insulin secretion indexes (ΔC30), or insulinogenic indexes (ΔC30/ΔG30 and ΔC120/ΔG120). Considering that the difference in baseline characteristics between the two surgical procedure groups was significant, we excluded surgical procedures when performing the binary logistic regression. Taking age, duration, BMI, FPG, and C-peptide AUC into the binary regression, the results showed that C-peptide AUC and age were independent predictors of short-term T2D remission after MS (Table 3).

### 3.3. Predictors of Prolonged T2D Remission

Among 72 patients who underwent surgery for more than 4 years, 50 of them (69.44%) underwent long-term follow-up after the surgery. There were 19 (38.00%) patients who remained in prolonged remission; however, 5 patients who achieved remission at 1-2 years after surgery experienced T2D recurrence in the long term. Univariate analysis showed that FPG, HbA1c, and C-peptide AUC values were factors affecting T2D remission 4-6 years after surgery, while there was no significant difference in age, sex, duration, surgical procedures, BMI, lipid profiles, FCP, ΔC30, ΔC120, ΔC30/ΔG30, ΔC120/ΔG120, HOMA-*β* (CP, DM), HOMA-IR (CP), presurgery insulin use, and UmAlb (Table 4). Binary regression analysis showed that level of C-peptide AUC and FPG values was closely associated with long-term T2D remission after MS (Table 5).

### 3.4. Optimal Cutoff Values for C-Peptide AUC

**Figure 1:**
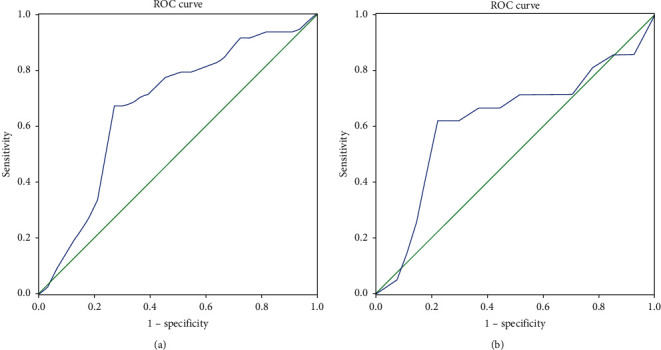
ROC curve of C-peptide AUC in predicting T2D remission. (a) The area under the ROC curve for prediction of short-term T2D remission using C-peptide AUC was 0.674 (specificity: 67.3%; sensitivity: 75.8%). (b) The area under the ROC curve for prediction of long-term T2D remission using C-peptide AUC was 0.623 (specificity: 61.9%; sensitivity: 81.5%).

Through the analysis of remission predictors, we noticed that C-peptide AUC has independent prognostic significance in T2D remission after MS, both in the short term and in the long term. To further explore the predictive ability of C-peptide AUC in T2D remission, we drew ROC curves (Figure 1). The optimal cutoff values for C-peptide AUC in predicting T2D remission were 30.93 ng/ml, with areas under the ROC curves of 0.674 and 0.623 in the short term and long term, respectively (Table 6).

### 3.5. ACF Scoring System

Integrating our results above and the data published by other researchers, we hypothesized that a multidimensional grading system that assessed age, FPG, and C-peptide AUC would better categorize the illness and predict the success of MS. The scoring system was abbreviated as ACF according to the initials of the indexes (Table 7). Applying the ACF score in this cohort (patients lacking C-peptide AUC values were excluded), we found that patients with a greater ACF score had a higher remission rate than other patients (Table 8). Patients with ACF scores ranging from 6 to 9 had short-term remission rates of 100.00% and prolonged remission rates of 80.00%, and those with scores ranging from 2 to 5 had remission rates of 61.11% and 50.00% in the short and long term, respectively, while patients who scored 0 had the lowest remission rate (28.00% in the short term and 10.53% in the long term).

To verify the practical ability of the ACF scoring system, we also applied other scoring systems to this cohort. The distribution of ABCD, DiaRem, and IMS scores of the studied patients is shown in Table 8. We found that the patients were more uniformly distributed using the ACF score than using the IMS score. Although the ABCD and DiaRem scores distributed patients better than the IMS score, the difference in remission rate among groups was not significant like the ACF score.

### 3.6. ROC Curve Analysis

**Figure 2:**
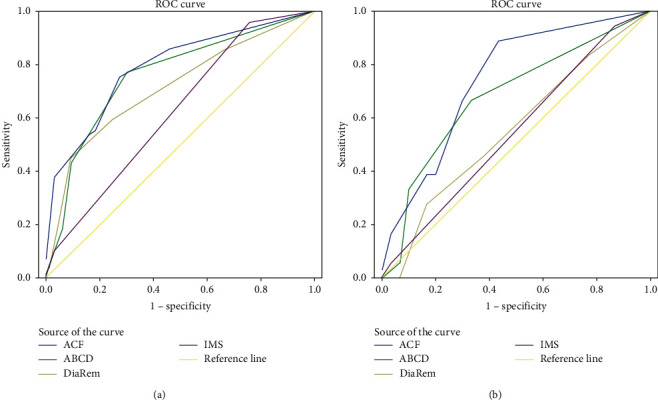
ROC curve analysis of ACF, ABCD, Diarem, and IMS scores. (a) In the short-term T2D remission prediction, the area under the ROC curves using the ACF score (the blue line) is 0.793, *P* < 0.001^*∗*^; the area under the ROC curves using the ABCD score (the green line) is 0.762, *P* < 0.001^*∗*^; the area under the ROC curves using the DiaRem score (the brown line) is 0.715, *P*=0.001^*∗*^; and the area under the ROC curves using the IMS score (the purple line) is 0.625, *P*=0.056. (b) In the long-term T2D remission prediction, the area under the ROC curves using the ACF score (the blue line) is 0.748, *P*=0.004^*∗*^; the area under the ROC curves using the ABCD score (the green line) is 0.681, *P*=0.038^*∗*^; the area under the ROC curves using the DiaRem score (the brown line) is 0.556, *P*=0.086; and the area under the ROC curves using the IMS score (the purple line) is 0.547, *P*=0.086.

On applying the ROC analysis (Figure 2), we could see that the ACF score had the maximum AUC in both the short-term and the long-term T2D remission prediction. The differences between the ACF and IMS score in the short-term prediction and the difference between ACF and DiaRem or IMS scores in the long-term prediction were significant (Table 9).

## 4. Discussion

Metabolic surgery has been now widely accepted as a method of treatment for diabetes that is superior to medical intervention for weight loss and diabetes remission [37, 38]. Recently, Chinese scholars reported T2D remission rates after MS of approximately 63.6–73.2% [20, 39, 40]. The remission rate of T2D after MS varied widely because of different follow-up lengths, participant features, severity of T2D, and so on. In this study, 60.92% (53 of 87) of patients experienced T2D resolution in 1-2 years following MS, and five of them had T2D recurrence thereafter. Of the 69.44% (50 of 72) of patients completing 4–6 years of follow-up, 38.00% (19 of 50) remained in prolonged remission.

It is worth noting that patients with T2D are not all in the same situation, and the optimal candidates for MS should be identified. Although predictive factors for T2D remission after MS in Asian populations have already been published by other researchers, including young age, high BMI, short duration, lower levels of FPG and HbA1c, higher C-peptide concentration, and preoperative insulin use [18, 41–43], values of *β*-cell function, especially comprehensive indexes reflecting *β*-cell function in T2D remission, were lacking. Progressive pancreatic *β*-cell failure is a natural course of diabetes and East Asian patients have been proved to have a worse *β*-cell function at the beginning of T2D. Focusing on the value of residual *β*-cell function, we explored the remission predictors of T2D remission after MS in mainland Chinese patients.

In what concerns *β*-cell function indexes, univariate regression showed that patients with higher FCP, higher ΔC120, higher HOMA-*β* (CP, DM), higher HOMA-IR (CP), and higher C-peptide AUC levels (grouped by median) have a much higher short-term remission rate than those in the lower groups, which indicates that *β*-cell function indexes play important roles in predicting short-term T2D remission after MS. Among them, the difference in C-peptide AUC is the most significant (*P* < 0.001). In the long-term follow-up, only patients with higher C-peptide AUC have a significant higher remission rate than those in the lower group, while there was no significant difference in the remission rate among groups classified by other *β*-cell function indexes. Patients with C-peptide AUC >30.93 ng/ml have a 2-fold possibility of T2D remission both in the short term (80.93% vs. 39.02%) and in the long term (55.56% vs. 26.67%). Moreover, as it was the only factor that showed significance in both the short- and long-term binary regression, we had to acknowledge the independent prognostic significance. Furthermore, we explored the optimal cutoff point for C-peptide AUC in predicting T2D remission. ROC curves showed that the optimal cutoff value of C-peptide AUC in prediction was 30.93 ng/ml, with a specificity of 67.3% and sensitivity of 75.8% in the short-term T2D remission and with a specificity of 61.9% and sensitivity of 81.5% in the long-term T2D remission, respectively. The area under the ROC curves (0.674 and 0.623 for the short- and long-term, respectively) had a moderate ability to predict remission. It has been demonstrated that C-peptide AUC has an association with higher T2D remission rate in Western patients whose average BMIs were higher than 44 kg/m^2^ in the short-term follow-up [23, 34]. In the present study, we identified that C-peptide AUC is an independent predictor of T2D remission after MS in Chinese patients both in the short term and in the long term.

Some studies verified that age plays a role in T2D remission prediction after MS [28, 41]. In the present study, binary logistic regression showed that age younger than 40 years facilitates short-term T2D remission, which confirms that it is an independent predictor of short-term T2D remission after MS. In the long-term follow-up, younger patients (age < 40 years) had a higher remission rate than the older patients (*P*=0.274).

Regarding FPG, there were inconsistencies among studies. The level of fasting glucose value was found to be associated with T2D remission in different reports [20, 34, 44]. The present study showed that patients with elevated level of FPG values had lower remission rates both in the short term and long term, and the significance remained in the long-term binary regression.

The other parameters that were significant in the univariate regression, including BMI, surgical procedures, the duration of T2D, HbA1c, and presurgical insulin usage, have all been reported by other studies [44–47]. In our study, the remission rate of patients with higher BMI was significantly higher in 1-2 years after surgery. However, the difference was not significant in the binary regression nor in the long-term remission prediction. Studies have shown that Asian patients with BMIs below 35 kg/m^2^ obtain successful glycemic control after bariatric surgery [19, 48]. Therefore, we speculated that BMI may not be the most important factor in T2D remission prediction. Besides, some studies have shown that weight loss after MS may be a predictive factor for T2D remission in addition to preoperative BMI [18, 49]. In the present study, we mainly discussed the predictive value of preoperation factors, so we did not include the EWL% or TWL%. In this cohort, over 95% of the patients who chose GB did so before 2014, while approximately 70% of patients underwent SG thereafter. Therefore, different enrolment standards and advances in surgical techniques and postsurgery management may have contributed to the different remission rates between the two groups. In addition, the preoperative data showed a significant difference between the two groups, and only a minority of patients underwent SG (28.7%). Therefore, we did not include surgical procedures in the binary logistic regression to minimize the deviation. A shorter duration of T2D was shown to be significantly associated with a higher remission rate in the short-term follow-up. And, we verified that longer T2D duration was correlated with worse *β*-cell function (FCP, ΔC30/ΔG30, and C-peptide AUC) and lower insulin resistance (HOMA-IR (CP)). And, presurgery insulin use was found to be associated with only short-term remission. Longer T2D duration and presurgery insulin use both partly imply worse *β*-cell function and thus may be corrected by other indexes in the binary logistic analysis. Additionally, HbA1c failed to show significance in the binary regression. No association between T2D remission and sex, lipid biomarkers, or indicators reflecting microvascular diseases (UmAlb) was found herein.

To select the patients best suited to MS, identifying preoperative predictors of T2D remission can lead to improved outcomes, and researchers have proposed several prediction models, such as the ABCD, DiaRem, and IMS scoring systems. Interestingly, *β*-cell function was not included in the scores except those for the ABCD. According to our study, *β*-cell function, especially C-peptide AUC, is a decisive predictive factor. Therefore, we tried to establish our own scoring system with regard to the regression analysis. We included three indexes in the scoring system according to regression analysis: age, FPG, and C-peptide AUC. Patients with a greater ACF score had a higher remission rate in both the short term and long term. We classified patients into three groups according to the ACF score. Those with scores ranging from 6–9 had the highest remission rate (100.00% and 80.00% in the short term and long term, respectively), and patients in the second group, whose score ranged from 2–5, achieved a remission rate of 61.11% in the short term and 50.00% in the long term. The patients with the lowest scores had the lowest remission rates (28.00% in the short term and 10.53% in the long term). Then, we compared our scoring system with others to verify the prognostic accuracy. The ACF score showed more significance in predicting the remission rate among the groups compared with the ABCD and DiaRem scores and a more uniform distribution compared with the IMS score. According to the ROC curves, the ACF score had the maximum AUC both in the short- and long-term remission prediction. The reason why the IMS and DiaRem scores did not show excellent predictive abilities in this cohort may be the different race of the patients and significant differences in baseline characteristics. Both of the original and validated data in those two studies came from severely obese Caucasians, with BMIs higher than 45 kg/m^2^, while our study was based on Chinese participants with an average BMI of 31.29 ± 6.51 kg/m^2^. We noticed that when compared with the ABCD score, our study showed superiority in grading of remission rates among groups and a larger AUC. The ABCD scoring system included FCP as an index of *β*-cell function, and our study showed that C-peptide AUC is a superior predictor than FCP in T2D remission. Another difference between the ACF and ABCD scores was that the latter included T2D duration, which was not considered as an independent predictive factor in this cohort.

This is the first prediction model of T2D remission after MS based on a mainland Chinese population, and it notably includes C-peptide AUC, an index of residual *β*-cell function, for choosing the optimal metabolic procedure among the two common surgical procedures. The ACF scoring system showed excellent predictive value in the present study. Undoubtedly, it still needs to be validated externally to verify its feasibility.

The limitations of this study include being a single-center study and having a relatively small number of study subjects. These factors precluded us from a more complicated statistical analysis and might also have weakened the statistical power to a certain degree. However, the ACF score is simple to calculate, which makes it a practical tool and with potentially widespread applicability.

## 5. Conclusions

In summary, the present study has shown that presurgery age, C-peptide AUC, and FPG are independent predictors of T2D remission after MS. Among these, C-peptide AUC plays a decisive role in both short- and long-term remission prediction, and the optimal cutoff value for C-peptide AUC in predicting T2D remission was 30.93 ng/ml, with moderate predictive values. The ACF score is a simple reliable system that can predict T2D remission among Chinese patients.

## Figures and Tables

**Table 1:** Characteristics of the patients at baseline.

Parameters	Overall (*N* = 87)		GB (*N* = 62)		SG (*N* = 25)		*P* value
	*N*	Mean ± SD (median)	*N*	Mean ± SD (median)	*N*	Mean ± SD (median)	
Age (yr)	87	44.2 ± 11.2 (45)	62	46.8 ± 9.6 (47)	25	37.7 ± 12.5 (35)	<0.001^*∗*^
Duration (yr)	87	6.4 ± 4.8 (6)	62	6.7 ± 4.7 (6.5)	25	5.7 ± 5.0 (5)	0.286
BMI (kg/m^2^)	87	31.29 ± 6.51 (29.41)	62	28.96 ± 4.84 (28.04)	25	37.09 ± 6.57 (35.64)	<0.001^*∗*^
SBP (mmHg)	87	136.1 ± 16.5 (135)	62	135.9 ± 16.8 (135)	25	136.5 ± 15.9 (135)	0.884
DBP (mmHg)	87	86.1 ± 11.7 (81)	62	85.6 ± 10.7 (80)	25	87.4 ± 14.0 (83)	0.543
TG (mmol/L)	87	2.63 ± 2.40 (1.93)	62	2.67 ± 2.50 (1.91)	25	2.53 ± 2.19 (1.93)	0.870
CHOL (mmol/L)	87	4.85 ± 1.08 (4.87)	62	4.87 ± 1.12 (4.87)	25	4.79 ± 1.06 (4.89)	0.974
HDL (mmol/L)	87	1.11 ± 0.26 (1.09)	62	1.14 ± 0.27 (1.13)	25	1.03 ± 0.24 (1.01)	0.030^*∗*^
LDL (mmol/L)	87	2.66 ± 0.84 (2.64)	62	2.67 ± 0.82 (2.67)	25	2.62 ± 0.91 (2.62)	0.955
FPG (mmol/L)	87	7.61 ± 2.22 (7.26)	62	7.57 ± 1.97 (7.39)	25	7.73 ± 2.78 (7.17)	0.760
HbA1c (%)	87	8.01 ± 1.70 (7.9)	62	8.18 ± 1.64 (7.9)	25	7.58 ± 1.80 (7.8)	0.108
FCP (ng/mL)	87	2.68 ± 1.84 (2.63)	62	2.25 ± 1.88 (1.66)	25	3.73 ± 1.22 (3.38)	<0.001^*∗*^
ΔC30 (ng/mL)	82	1.58 ± 1.70 (1.01)	59	1.24 ± 1.53 (0.75)	23	2.44 ± 1.85 (1.62)	0.001^*∗*^
ΔC120 (ng/mL)	87	4.57 ± 6.80 (3.08)	62	3.98 ± 7.74 (2.51)	25	6.04 ± 3.25 (5.38)	<0.001^*∗*^
ΔC30/ΔG30 (*μg*/mmol)	82	0.36 ± 0.46 (0.27)	59	0.28 ± 0.39 (0.15)	23	0.58 ± 0.55 (0.44)	0.003^*∗*^
ΔC120/ΔG120 (*μg*/mmol)	87	0.71 ± 1.35 (0.42)	62	0.53 ± 1.07 (0.31)	25	1.16 ± 1.84 (0.73)	<0.001^*∗*^
HOMA-*β* (CP, DM)(%)	87	72.48 ± 59.97 (52.71)	62	57.80 ± 48.95 (41.14)	25	108.87 ± 69.71 (94.97)	<0.001^*∗*^
HOMA-IR (CP)	87	2.49 ± 1.93 (2.08)	62	2.14 ± 1.98 (1.43)	25	3.35 ± 1.55 (3.06)	<0.001^*∗*^
C-peptide AUC (ng/ml)	82	38.90 ± 38.89 (30.93)	59	33.22 ± 36.52 (24.95)	23	53.47 ± 20.06 (52.77)	<0.001^*∗*^
UmAlb (mg/L)	74	89.85 ± 142.95 (31.95)	56	71.32 ± 97.20 (31.50)	18	147.49 ± 229.20 (34.50)	0.545

Data are presented as the mean ± standard deviation (median). SBP: systolic blood pressure; DBP: diastolic blood pressure; TG: triglycerides; CHOL: cholesterol; HDL: high-density lipoprotein; LDL: low-density lipoprotein; FPG: fasting plasma glucose; FCP: fasting C-peptide; C-peptide AUC: C-peptide area under the curve; UmAlb: urinary microalbumin. ^*∗*^*P* < 0.05.

**Table 2:** Univariate analysis of remission predictors 1-2 years after surgery.

	Remission group (*N* = 53)	Nonremission group (*N* = 34)	*P* value
Age (yr)			<0.001^*∗*^
<40	22 (91.67%)	2 (8.33%)	
≥40	31 (49.21%)	32 (50.79%)	
Gender			0.101
Female	13 (48.15%)	14 (51.85%)	
Male	40 (66.67%)	20 (33.33%)	
Duration (yr)			0.011^*∗*^
≤5	32 (74.42%)	11 (25.58%)	
>5	21 (47.73%)	23 (52.27%)	
Surgical procedures			<0.001^*∗*^
Sleeve gastrectomy	23 (92.00%)	2 (8.00%)	
Gastric bypass	30 (48.39%)	32 (51.62%)	
BMI (kg/m^2^)			<0.001^*∗*^
<27.5	12 (41.38%)	17 (58.62%)	
27.5–32.5	20 (55.56%)	16 (44.44%)	
≥32.5	21 (95.45%)	1 (4.55%)	
TG (mmol/L)			0.493
≥1.7	32 (58.18%)	23 (41.82%)	
<1.7	21 (65.63%)	11 (34.37%)	
CHOL (mmol/L)			0.426
≥4.14	40 (63.49%)	23 (36.51%)	
<4.14	13 (54.17%)	11 (45.83%)	
LDL (mmol/L)			0.534
≥2.59	26 (57.78%)	19 (42.22%)	
<2.59	27 (64.29%)	15 (35.71%)	
HDL (mmol/L)			0.279
>1.29	11 (73.33%)	4 (26.67%)	
≤1.29	42 (58.33%)	30 (41.67%)	
FPG (mmol/L)			0.099
<7.0	25 (71.43%)	10 (28.57%)	
≥7.0	28 (53.85%)	24 (46.15%)	
HbA1c (%)			0.270
<6.5	13 (76.47%)	4 (25.53%)	
6.5 ≤ HbA1c < 9	27 (60.00%)	18 (40.00%)	
≥9	13 (52.00%)	12 (48.00%)	
FCP (ng/mL)	(*N* = 53)	(*N* = 34)	0.011^*∗*^
≤2.63	21 (47.73%)	23 (52.27%)	
>2.63	32 (74.42%)	11 (25.58%)	
ΔC30 (ng/mL)	(*N* = 49)	(*N* = 33)	0.499
<1.01	23 (56.10%)	18 (43.90%)	
≥1.01	26 (63.41%)	15 (36.59%)	
ΔC120 (ng/mL)	(*N* = 53)	(*N* = 34)	0.035^*∗*^
≤3.08	22 (50.00%)	22 (50.00%)	
>3.08	31 (72.09%)	12 (27.91%)	
ΔC30/ΔG30 (*μ*g/mmol)	(*N* = 49)	(*N* = 33)	0.499
≤0.27	23 (56.10%)	18 (43.90%)	
>0.27	26 (63.41%)	15 (36.59%)	
ΔC120/ΔG120 (*μ*g/mmol)	(*N* = 53)	(*N* = 34)	0.095
≤0.42	23 (52.27%)	21 (47.73%)	
>0.42	30 (69.77%)	13 (30.22%)	
HOMA-*β* (CP, DM) (%)	(*N* = 53)	(*N* = 34)	0.003^*∗*^
≤52.71	20 (45.45%)	24 (54.55%)	
>52.71	33 (76.74%)	10 (23.26%)	
HOMA-IR (CP)	(*N* = 53)	(*N* = 34)	0.011^*∗*^
≤2.08	21 (47.73%)	23 (52.27%)	
>2.08	32 (74.42%)	11 (25.58%)	
C-peptide AUC (ng/ml)	(*N* = 49)	(*N* = 33)	<0.001^*∗*^
≤30.93	16 (39.02%)	25 (60.98%)	
>30.93	33 (80.49%)	8 (19.51%)	
UmAlb(mg/L)	(*N* = 42)	(*N* = 32)	0.687
<20	15 (60.00%)	10 (40.00%)	
≥20	27 (55.10%)	22 (44.90%)	
Preoperative insulin use			<0.001^*∗*^
Yes	20 (43.48%)	26 (56.52%)	
No	33 (80.49%)	8 (19.52%)	

^∗^ *P* < 0.05; indexes of islet function ((FCP, ΔC30, ΔC120, ΔC30/ΔG30, ΔC120/ΔG120, HOMA-*β* (CP, DM), HOMA-IR (CP), and C-peptide AUC) were grouped by median.

**Table 3:** Binary logistic regression of short-term remission predictors.

	*P* value	OR	CI
C-peptide AUC (ng/ml)	0.002^*∗*^	4.530	1.581–12.983
≤30.93	Reference		
>30.93			
Age (yr)	0.012^*∗*^	7.705	1.567–37.874
≥40	Reference		
<40			
BMI (kg/m^2^)	0.136		
<27.5	Reference		
27.5–32.5			
≥27.5			
Duration (yr)	0.097		
≤5	Reference		
>5			
FPG (mmol/L)	0.279		
<7.0	Reference		
≥7.0			

^*∗*^ *P* < 0.05.

**Table 4:** Univariate analysis of prolonged remission predictors.

	Remission group (*N* = 19)	Nonremission group (*N* = 31)	*P* value
Age (yr)			0.274
<40	4 (66.67%)	2 (33.33%)	
≥40	15 (34.09%)	29 (65.91%)	
Gender			0.085
Female	4 (22.22%)	14 (77.78%)	
Male	15 (47.87%)	17 (53.13%)	
Duration (yr)			0.121
≤5	11 (50.00%)	11 (50.00%)	
>5	8 (28.57%)	20 (71.43%)	
Surgical procedures			0.759
Sleeve gastrectomy	2 (66.67%)	1 (33.33%)	
Gastric bypass	20 (40.00%)	30 (60.00%)	
BMI (kg/m^2^)			0.057
<27.5	6 (28.57%)	15 (71.43%)	
27.5–32.5	8 (34.78%)	15 (65.22%)	
≥32.5	5 (83.33%)	1 (16.67%)	
TG (mmol/L)			0.923
≥1.7	12 (37.50%)	20 (62.50%)	
<1.7	7 (38.89%)	11 (61.11%)	
CHOL (mmol/L)			0.280
≥4.14	15 (42.86%)	20 (57.14%)	
<4.14	4 (26.67%)	11 (73.33%)	
LDL (mmol/L)			0.564
≥2.59	8 (33.33%)	16 (66.67%)	
<2.59	11 (42.31%)	15 (57.69%)	
HDL (mmol/L)			0.115
>1.29	6 (66.67%)	3 (33.33%)	
≤1.29	13 (31.71%)	28 (68.29%)	
FPG (mmol/L)			0.043^*∗*^
<7.0	11 (55.00%)	9 (45.00%)	
≥7.0	8 (26.67%)	22 (73.33%)	
HbA1c (%)			0.049^*∗*^
<6.5	2 (28.57%)	5 (71.43%)	
6.5 ≤ HbA1c < 9	15 (51.72%)	14 (48.28%)	
≥9	2 (14.29%)	12 (85.71%)	
FCP (ng/mL)	(*N* = 19)	(*N* = 31)	0.923
≤2.63	12 (37.00%)	20 (62.50%)	
>2.63	7 (38.89%)	11 (61.11%)	
ΔC30 (ng/mL)	(*N* = 18)	(*N* = 30)	0.823
<1.01	9 (36.00%)	16 (64.00%)	
≥1.01	9 (39.13%)	14 (60.87%)	
ΔC120 (ng/mL)	(*N* = 19)	(*N* = 31)	0.777
≤3.08	13 (39.39%)	20 (60.61%)	
>3.08	6 (35.29%)	11 (64.71%)	
ΔC30/ΔG30 (*μ*g/mmol)	(*N* = 18)	(*N* = 30)	0.654
≤0.27	9 (34.62%)	17 (65.38%)	
>0.27	9 (40.91%)	13 (59.09%)	
ΔC120/ΔG120 (*μ*g/mmol)	(*N* = 19)	(*N* = 31)	0.812
≤0.42	11 (36.67%)	19 (63.33%)	
>0.42	8 (40.00%)	12 (60.00%)	
HOMA-*β* (CP, DM) (%)	(*N* = 19)	(*N* = 31)	0.233
≤52.71	9 (31.03%)	20 (68.97%)	
>52.71	10 (47.62%)	11 (52.38%)	
HOMA-IR (CP)	(*N* = 19)	(*N* = 31)	0.640
≤2.08	11 (35.48%)	20 (64.52%)	
>2.08	8 (42.11%)	11 (57.89%)	
C-peptide AUC (ng/ml)	(*N* = 18)	(*N* = 30)	0.045^*∗*^
≤30.93	8 (26.67%)	22 (73.33%)	
>30.93	10 (55.56%)	8 (44.44%)	
UmAlb (mg/L)	(*N* = 16)	(*N* = 28)	0.596
<20	6 (46.15%)	7 (53.85%)	
≥20	10 (32.66%)	21 (67.74%)	
Preoperative insulin use			0.405
Yes	10 (33.33%)	20 (66.67%)	
No	9 (45.00%)	11 (55.00%)	

^*∗*^ *P* < 0.05; indexes of islet function (FCP, ΔC30, ΔC120, ΔC30/ΔG30, ΔC120/ΔG120, HOMA-*β* (CP, DM), HOMA-IR (CP), and C-peptide AUC) were grouped by median.

**Table 5:** Binary logistic regression of prolonged remission predictors.

	*P* value	OR	CI
C-peptide AUC (ng/ml)	0.002	8.139	1.962–33.762
≤30.93	Reference		
>30.93			
FPG (mmol/L)	0.026^*∗*^	4.517	1.113–18.333
≥7.0	Reference		
<7.0			
Gender	0.078		
Female	Reference		
Male			
HbA1c (%)	0.976		
<6.5	Reference		
6.5 ≤ HbA1c < 9			
≥9			
BMI (kg/m^2^)	0.606		
<27.5	Reference		
27.5–32.5			
≥32.5			

^*∗*^ *P* < 0.05.

**Table 6:** ROC analysis of C-peptide AUC in remission prediction.

	Optimal cutoff value (ng/ml)	Sensitivity (%)	Specificity (%)	Youden's index	AUC
Short-term remission	30.93	67.3	75.8	0.431	0.674
Prolonged remission	30.93	61.9	81.5	0.434	0.623

AUC: area under the ROC curve.

**Table 7:** ACF score.

Factor	Score
Age (yr)	
≥40	0
<40	4
C-peptide AUC (ng/ml)	
≤30.93	0
>30.93	3
FPG (mmol/L)	
≥7.0	0
<7.0	2
Total	0–9

**Table 8:** Predictive ability of scores.

Model	Total points	Short-term data (*N* = 82)			Long-term data (*N* = 48)		
		Remission (*N*)	Nonremission (*N*)	Remission rate (%)	Remission (*N*)	Nonremission (*N*)	Remission rate (%)
ACF	0	7	18	28.00	2	17	10.53
	2–5	22	14	61.11	12	12	50.00
	6–9	20	0	100.00	4	1	80.00

ABCD	0–2	11	23	32.35	6	20	23.08
	3-4	17	7	70.83	6	7	46.15
	5-6	12	1	92.31	5	1	83.33
	7-8	8	2	80.00	1	2	33.33
	9-10	1	0	100.00	—	—	—

DiaRem	0–2	7	11	38.89	3	7	30.00
	3–7	13	14	48.15	7	12	36.84
	8–12	7	5	58.33	3	6	33.33
	13–17	16	2	88.89	5	3	62.50
	18–22	6	11	85.71	0	2	0.00

IMS	25	2	8	20.00	1	4	20.00
	25–95	42	24	63.64	16	25	39.02
	≥95	5	1	83.33	1	1	50.00

**Table 9:** Comparison of ROC analysis of different models.

	Model	AUC	*P* value	95% CI
Short-term data	ACF	0.793	<0.001^*∗*^	0.696–0.890
	ABCD	0.762	<0.001^*∗*^	0.655–0.869
	DiaRem	0.715	0.001^*∗*^	0.603–0.826
	IMS	0.625^§^	0.056	0.500–0.750

Long-term data	ACF	0.748	0.004^*∗*^	0.607–0.889
	ABCD	0.681	0.038^*∗*^	0.387–0.724
	DiaRem	0.556^§^	0.086	0.380–0.715
	IMS	0.547^§^	0.086	0.520–0.841

AUC: area under the ROC curve. ^§^Compared to ACF, *P* value of AUC <0.05. ^*∗*^*P* < 0.05.

## Data Availability

The data used to support the findings of this study are available from the corresponding author upon request.
